# Interaction Between Humans and Poultry, Rural Cambodia

**DOI:** 10.3201/eid1301.061014

**Published:** 2007-01

**Authors:** Sowath Ly, Maria D. Van Kerkhove, Davun Holl, Yves Froehlich, Sirenda Vong

**Affiliations:** * Pasteur Institute in Cambodia, Phnom Penh, Cambodia; † London School of Hygiene and Tropical Medicine, London, United Kingdom; ‡ Ministry of Agriculture, Forestry and Fisheries, Phnom Penh, Cambodia; § Food and Agriculture Organization of the United Nations, Phnom Penh, Cambodia

**Keywords:** Highly pathogenic avian influenza, H5N1, poultry handling behavior, human, Cambodia, dispatch

## Abstract

Because avian influenza H5N1 infection risks are associated with exposure to infected poultry, we conducted a knowledge, attitudes, and practices survey of poultry-handling behavior among villagers in rural Cambodia. Despite widespread knowledge of avian influenza and personal protection measures, most rural Cambodians still have a high level of at-risk poultry handling.

The circulation of the highly pathogenic H5N1 avian influenza (AI) strain throughout Asia since late 2003 (*1*), and more recently in Europe and Africa, has resulted in considerable concern for the potential of a new pandemic. In Cambodia, outbreaks of HPAI A/H5N1 infection were first reported in poultry in early 2004 (*2*). Since 2005, 6 human cases have occurred (100% fatal); the 2 most recent cases occurred in early 2006 (*3*,*4*).

Most Cambodians live in rural areas and raise animals for consumption (*2*), typically keeping poultry, swine, or cattle close to the home. Because H5N1 infection has been associated with exposure to infected poultry (*5*–*10*) and little is understood of the perceptions of rural farmers regarding AI (*11*), we conducted a knowledge, attitude, and practices survey of poultry handling in rural Cambodia to estimate the extent of interactions between humans and poultry, to understand practices in poultry handling among villagers, and to develop interventions designed to increase reports of poultry deaths and safe poultry handling.

## The Study

We conducted a 2-stage household based cluster survey (*12*) with a goal of 500 participants: 20 persons >15 years of age in each of 25 villages from Prey Veng and Kampong Cham Provinces. The sampling frame of eligible villages within these provinces were those located in H5N1 high-risk communes, as defined by the Food and Agriculture Organization of the United Nations training program for village animal health workers. The villages were selected with probability proportional to size. For the second stage, we randomly selected the first household within each village. Subsequently, households were selected by proximity until 20 eligible participants were enrolled in each cluster.

Verbal consent was obtained from all participants. All were interviewed by using a structured questionnaire designed to collect information on demographics, basic hygiene practices, quantity of poultry owned, poultry death reporting, practices when deaths occurred, knowledge and attitude of sick and dead poultry, and knowledge of AI.

**Figure 1:**
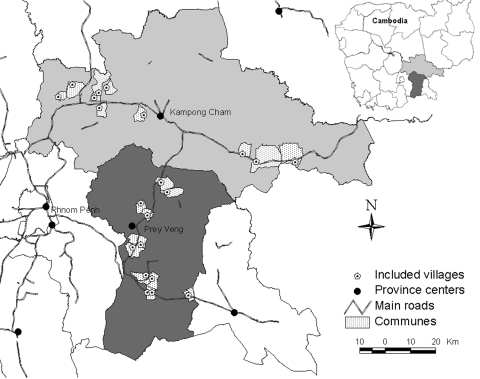
Distribution of selected communes in Kampong Cham and Prey Veng provinces, Cambodia, 2006.

Twenty-three villages were included in Kampong Cham (*11*) and Prey Veng (*12*) Provinces (Figure 1). Four hundred sixty respondents from 269 households completed the questionnaire. Most were women (60%), farmers (88%), and persons who had completed less than primary schooling (57%). The median number of household members was 5 (range 1–16), and 77% of all households included children <15 years of age.

**Figure 2:**
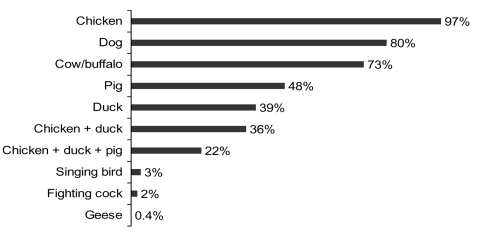
Proportions of animals raised in the household (n = 269).

Many households owned chickens (97%) and ducks (39%) (Figure 2), although the size of most poultry flocks was small (Table). Almost all poultry were free ranging (100% of chicken flocks; 96% of duck flocks), and mixing of the poultry with pigs and other domestic animals was common. Respondents reported that they use poultry feces for manure (77%), touch sick/dead poultry with bare hands (75%), eat poultry that died from illness (45%), eat wild birds (33%), let children touch sick/dead poultry with bare hands (20%), and gather dead wild birds for consumption (8%).

**Table:** Poultry raising and flock characteristics, rural Cambodia

Flock characteristics	Chickens (n = 261)	Ducks (n = 97)
Median number per flock (range)	10 (1–110)	6 (1–800)
1–25	83%	93%
26–50	15%	3%
51–100	2%	0%
>100	0.4%	4%
Animal age when raising begins (months)	0 (0–15)	0 (0–12)
Type of raising		
Free ranging	100%	96%
Raising purpose (noncumulative)		
Household needs	73%	70%
Meat for sale	54%	42%
Eggs for sale	1%	16%

During the previous 6 months, of the 260 households that owned poultry, 162 (62%) experienced poultry deaths; however, only 18 (7%) reported these deaths to local authorities. Half of the respondents (n = 231) believed that it was important to report any poultry deaths because the death may be due to AI (61%) or because the poultry owners may receive management advice from the village veterinarians (39%). Among these 231 respondents, many did not report poultry deaths because they did not know how (41%), were in the habit of not reporting poultry deaths (31%), believed they would have a problem selling poultry if they reported deaths (18%), did not know the risks of AI (7%), or feared poultry culling (5%). Among those respondents who did not believe reporting deaths was important, the reasons provided included the following: “the number of poultry deaths were too few” (62%), “poultry are not as important as cattle” (18%), “no help would be provided from veterinary staff or authorities” (13%), or “because mortality was similar to previous years” (7%). Of respondents that experienced poultry deaths, 62% buried or burned dead poultry, 53% prepared them for food, 22% threw away the dead poultry, 3% used them to feed other animals, and 2% prepared them for sale or gave them to their neighbors.

Participants had learned about AI from television (81%) and radio (78%). Thirty-one percent of respondents were able to describe AI symptoms in humans, and 72% believed that AI is a fatal disease among poultry that can be transmitted to humans. Most respondents believed it is unsafe to touch sick or dead poultry with bare hands (67%), eat wild birds (70%), let children touch sick or dead birds with bare hands (83%), and eat meat or eggs that are not fully cooked (86%). Sixty-one percent of respondents mentioned at least 1 of the recommended behavioral practices that protect against AI infection.

## Conclusions

General media reports about AI through radio and television broadcasts appear to have been effective at reaching rural people. However, despite high awareness and widespread knowledge about AI and personal protection measures, most rural Cambodians still often practice at-risk poultry handling. Anecdotally, we also reported that family members of H5N1-infected patients, who knew about AI risks, still prepared dead or sick poultry for household consumption during massive die-offs, because they observed that neighbors with the same behavior did not become sick (Institute Pasteur in Cambodia, unpub. data). These findings provide evidence that high awareness does not necessary lead to behavior change. Behavior change involves comprehensive and multidisciplinary intervention, which combines risk perception communication and feasible and practical recommendations, including economic considerations. We speculate that it is hardly feasible to sustain good poultry-handling practices if access to personal protective equipment is cost prohibitive, particularly when disease occurrence poultry die-offs are common. Further studies are needed to determine appropriate behavior change strategies in Cambodia.

We did find that many of the villagers were willing to report poultry deaths but did not know how. However, this finding should be interpreted in light of some limitations. We observed difficulties and frustrations among farmers whose flocks underwent culling after identification of H5N1 viruses in their flocks because compensation has not yet been approved by the government of Cambodia. In contrast, Thailand and Vietnam have introduced compensation along with the introduction of poultry vaccination in Vietnam and the reduction of backyard poultry ownership in Thailand in an effort to protect the commercial poultry industry. Thus, it is difficult to envision effective control strategies in Cambodia based exclusively on culling. Coincidentally, Vietnam has reported far fewer H5N1 outbreaks in poultry and humans since the introduction of the vaccination program, while Cambodia detected 4 outbreak sites in domestic poultry and 2 unrelated human cases in 2006. The real effect of a no-compensation policy on willingness to report poultry deaths needs to be assessed.

Not surprisingly, direct contact with poultry and poultry products was common among household members. Transmission of H5N1 from poultry to humans, even in circumstances in which human–poultry interactions are regular and intense has been limited; however, as the virus continue to circulate and evolve among poultry, bird-to-human transmission may increase. In this context, improvement in risky practices can only be achieved through relentless behavior change efforts. Because lack of knowledge does not appear to be a factor, intervention programs must include feasible options for resource-poor settings that have limited materials for personal protection (water, soap, rubber gloves, masks) and must offer farmers alternative methods to safely work with poultry on a daily basis.
